# Supplementary figures and images for: A tool to dissect heterotypic determinants of homotypic protein phase behavior
Source: bioRxiv. 2025 Jan 2:2025.01.01.631016. Preprint. [Version 1] doi: 10.1101/2025.01.01.631016 (PMC11722427; doi:10.1101/2025.01.01.631016)

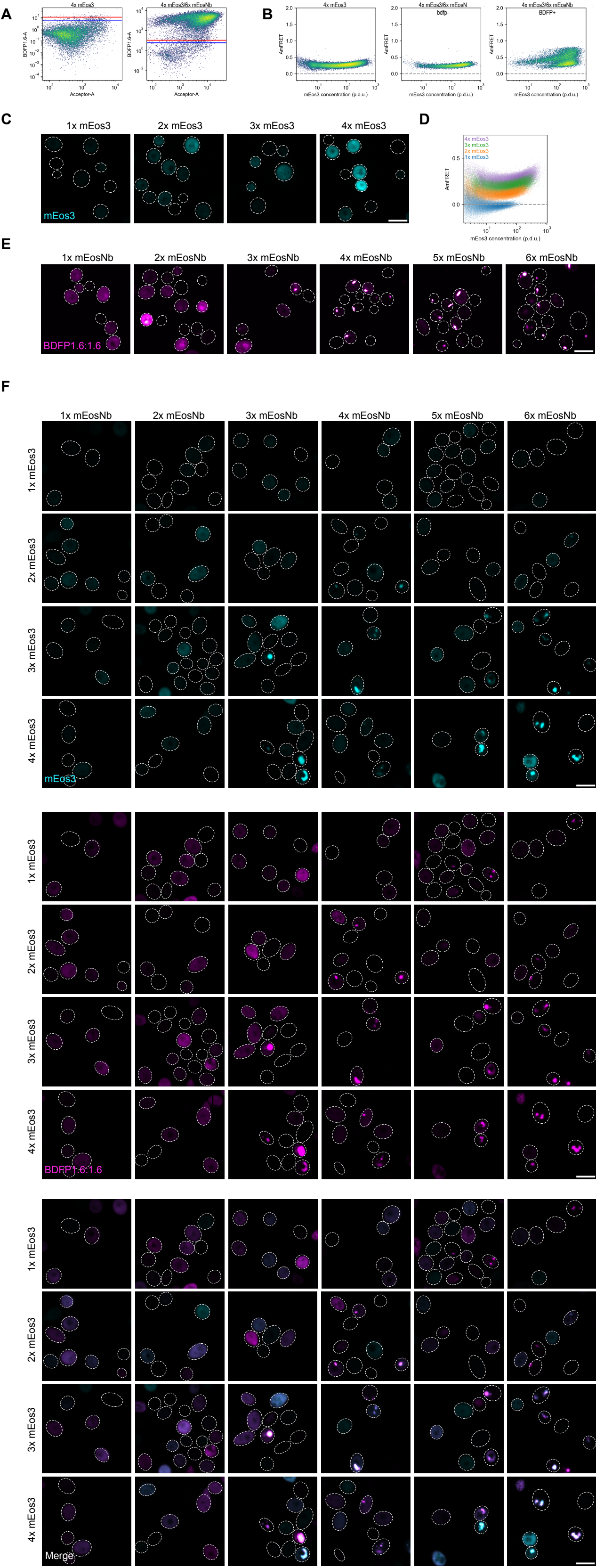

Supplement: Supplement 1 — Figure S1. Additional data supporting main figures. (a) The maximum BDFP1.6-A signal for BDFP1.6:1.6-negative (“bdfp-”) cells and the minimum BDFP1.6-A signal for BDFP1.6:1.6-positive (“BDFP+”) cells are shown in blue and red, respectively. Cells where the BDFP1.6-A signal was compensated to 0 or less are not included in these plots, but comprise 79990 cells for the control plot (86% of cells) and 16424 cells for 4x mEos3 with 6x mEosNb (39% of cells). (b) DAmFRET plots show that the bdfp- portion of the well with 4x mEos3 and 6x mEosNb is very similar to the control plot, while the BDFP+ portion shows differences due to expression of 6x mEosNb. (c). Representative confocal microscopy images of parental strains expressing query proteins 1–4x mEos3 displaying diffuse signal at all valencies and concentrations. (d) DAmFRET plot of multivalent mEos3 peptides revealing increased acceptor intensity and AmFRET as a function of valency, resulting from brighter intracellular signal and intramolecular FRET, respectively. (e) Representative confocal microscopy images of parental strains expressing query proteins 1–6x mEosNb, displaying an increase in puncta formation as concentration and valency increases. (f) Representative confocal images of pairwise combinations of multivalent mEos3 and mEosNb daughter cells as in Figure 3A, with brightness and contrast parameters adjusted the same within a fluorescence channel. Scale bars, 10μm. [file media-1.pdf]
