# Supplementary material for: A tool to dissect heterotypic determinants of homotypic protein phase behavior
Source: bioRxiv. 2025 Jan 2:2025.01.01.631016. Preprint. [Version 1] doi: 10.1101/2025.01.01.631016 (PMC11722427; doi:10.1101/2025.01.01.631016)
Supplement: Supplement 2 — Table S1. Plasmids and protein sequences used in this study. [file media-2.pdf]

Name	Insert	Source	Fusion protein sequence
rhx3912b	(HA)3	This study	MTRVSTEQRNLERHMDRILHSPGRHRDSPHSQEHRMGTFHLSLISNSCSPSPLCTRKLGP<br>FCYLLRSLLFCTNL
rhx5436	BDFP1.6:1.6-1xmEosNb	This study	MSMANREVVETLKELLADGEKRVQVAGVIGTNAAEVVKTAVSLLFQEYPELVSPGGCAYTTR<br>RYNMCVRDMNYFLRMCSYAIVAGGASVLDGRMLAGFRDTFNSLGIPLCPAARGIQLMKKI<br>VKEKLATAGMTNIAFVDEPFDYIARVISETEIGHGTGSTGSGSMANREVVETLKELLADGEKR<br>VQVAGVIGTNAAEVVKTAVSLLFQEYPELVSPGGCAYTTRRYNMCVRDMNYFLRMCSYAIV<br>AGGASVLDGRMLAGFRDTFNSLGIPLCPAARGIQLMKKIVKEKLATAGMTNIAFVDEPFDYI<br>ARVISETEIGGGGSMSRDQKMSQVQLQESGGGLVQAGGSLRLSCAASGNISQLVYMGWY<br>RQAPGKERELVAGITHGGITYYADSVKGRFTISRDNAKNTVYLQMNSLKPEDTAVYYCAAFQ<br>WRSDDVGYLNLGPLEYWGQGTQVTVSSKVSGA
rhx5437	BDFP1.6:1.6-2xmEosNb	This study	MSMANREVVETLKELLADGEKRVQVAGVIGTNAAEVVKTAVSLLFQEYPELVSPGGCAYTTR<br>RYNMCVRDMNYFLRMCSYAIVAGGASVLDGRMLAGFRDTFNSLGIPLCPAARGIQLMKKI<br>VKEKLATAGMTNIAFVDEPFDYIARVISETEIGHGTGSTGSGSMANREVVETLKELLADGEKR<br>VQVAGVIGTNAAEVVKTAVSLLFQEYPELVSPGGCAYTTRRYNMCVRDMNYFLRMCSYAIV<br>AGGASVLDGRMLAGFRDTFNSLGIPLCPAARGIQLMKKIVKEKLATAGMTNIAFVDEPFDYI<br>ARVISETEIGGGGSMSRDQKMSQVQLQESGGGLVQAGGSLRLSCAASGNISQLVYMGWY<br>RQAPGKERELVAGITHGGITYYADSVKGRFTISRDNAKNTVYLQMNSLKPEDTAVYYCAAFQ<br>WRSDDVGYLNLGPLEYWGQGTQVTVSSKVSGAGGGGSMSRDQKMSQVQLQESGGGLV<br>QAGGSLRLSCAASGNISQLVYMGWYRQAPGKERELVAGITHGGITYYADSVKGRFTISRDN<br>AKNTVYLQMNSLKPEDTAVYYCAAFQWRSDDVGYLNLGPLEYWGQGTQVTVSSKVSGA
rhx5438	BDFP1.6:1.6-3xmEosNb	This study	MSMANREVVETLKELLADGEKRVQVAGVIGTNAAEVVKTAVSLLFQEYPELVSPGGCAYTTR<br>RYNMCVRDMNYFLRMCSYAIVAGGASVLDGRMLAGFRDTFNSLGIPLCPAARGIQLMKKI<br>VKEKLATAGMTNIAFVDEPFDYIARVISETEIGHGTGSTGSGSMANREVVETLKELLADGEKR<br>VQVAGVIGTNAAEVVKTAVSLLFQEYPELVSPGGCAYTTRRYNMCVRDMNYFLRMCSYAIV<br>AGGASVLDGRMLAGFRDTFNSLGIPLCPAARGIQLMKKIVKEKLATAGMTNIAFVDEPFDYI<br>ARVISETEIGGGGSMSRDQKMSQVQLQESGGGLVQAGGSLRLSCAASGNISQLVYMGWY<br>RQAPGKERELVAGITHGGITYYADSVKGRFTISRDNAKNTVYLQMNSLKPEDTAVYYCAAFQ<br>WRSDDVGYLNLGPLEYWGQGTQVTVSSKVSGAGGGGSMSRDQKMSQVQLQESGGGLV<br>QAGGSLRLSCAASGNISQLVYMGWYRQAPGKERELVAGITHGGITYYADSVKGRFTISRDN<br>AKNTVYLQMNSLKPEDTAVYYCAAFQWRSDDVGYLNLGPLEYWGQGTQVTVSSKVSGAG<br>GGGSMSRDQKMSQVQLQESGGGLVQAGGSLRLSCAASGNISQLVYMGWYRQAPGKER<br>ELVAGITHGGITYYADSVKGRFTISRDNAKNTVYLQMNSLKPEDTAVYYCAAFQWRSDDVG<br>YLNLGPLEYWGQGTQVTVSSKVSGA
rhx5439	BDFP1.6:1.6-4xmEosNb	This study	MSMANREVVETLKELLADGEKRVQVAGVIGTNAAEVVKTAVSLLFQEYPELVSPGGCAYTTR<br>RYNMCVRDMNYFLRMCSYAIVAGGASVLDGRMLAGFRDTFNSLGIPLCPAARGIQLMKKI<br>VKEKLATAGMTNIAFVDEPFDYIARVISETEIGHGTGSTGSGSMANREVVETLKELLADGEKR<br>VQVAGVIGTNAAEVVKTAVSLLFQEYPELVSPGGCAYTTRRYNMCVRDMNYFLRMCSYAIV<br>AGGASVLDGRMLAGFRDTFNSLGIPLCPAARGIQLMKKIVKEKLATAGMTNIAFVDEPFDYI<br>ARVISETEIGGGGSMSRDQKMSQVQLQESGGGLVQAGGSLRLSCAASGNISQLVYMGWY<br>RQAPGKERELVAGITHGGITYYADSVKGRFTISRDNAKNTVYLQMNSLKPEDTAVYYCAAFQ<br>WRSDDVGYLNLGPLEYWGQGTQVTVSSKVSGAGGGGSMSRDQKMSQVQLQESGGGLV<br>QAGGSLRLSCAASGNISQLVYMGWYRQAPGKERELVAGITHGGITYYADSVKGRFTISRDN<br>AKNTVYLQMNSLKPEDTAVYYCAAFQWRSDDVGYLNLGPLEYWGQGTQVTVSSKVSGAG<br>GGGSMSRDQKMSQVQLQESGGGLVQAGGSLRLSCAASGNISQLVYMGWYRQAPGKER<br>ELVAGITHGGITYYADSVKGRFTISRDNAKNTVYLQMNSLKPEDTAVYYCAAFQWRSDDVG<br>YLNLGPLEYWGQGTQVTVSSKVSGAGGGGSMSRDQKMSQVQLQESGGGLVQAGGSLRL<br>SCAASGNISQLVYMGWYRQAPGKERELVAGITHGGITYYADSVKGRFTISRDNAKNTVYLQ<br>MNSLKPEDTAVYYCAAFQWRSDDVGYLNLGPLEYWGQGTQVTVSSKVSGA
rhx5440	BDFP1.6:1.6-5xmEosNb	This study	MSMANREVVETLKELLADGEKRVQVAGVIGTNAAEVVKTAVSLLFQEYPELVSPGGCAYTTR<br>RYNMCVRDMNYFLRMCSYAIVAGGASVLDGRMLAGFRDTFNSLGIPLCPAARGIQLMKKI<br>VKEKLATAGMTNIAFVDEPFDYIARVISETEIGHGTGSTGSGSMANREVVETLKELLADGEKR<br>VQVAGVIGTNAAEVVKTAVSLLFQEYPELVSPGGCAYTTRRYNMCVRDMNYFLRMCSYAIV<br>AGGASVLDGRMLAGFRDTFNSLGIPLCPAARGIQLMKKIVKEKLATAGMTNIAFVDEPFDYI<br>ARVISETEIGGGGSMSRDQKMSQVQLQESGGGLVQAGGSLRLSCAASGNISQLVYMGWY<br>RQAPGKERELVAGITHGGITYYADSVKGRFTISRDNAKNTVYLQMNSLKPEDTAVYYCAAFQ<br>WRSDDVGYLNLGPLEYWGQGTQVTVSSKVSGAGGGGSMSRDQKMSQVQLQESGGGLV<br>QAGGSLRLSCAASGNISQLVYMGWYRQAPGKERELVAGITHGGITYYADSVKGRFTISRDN<br>AKNTVYLQMNSLKPEDTAVYYCAAFQWRSDDVGYLNLGPLEYWGQGTQVTVSSKVSGAG<br>GGGSMSRDQKMSQVQLQESGGGLVQAGGSLRLSCAASGNISQLVYMGWYRQAPGKER<br>ELVAGITHGGITYYADSVKGRFTISRDNAKNTVYLQMNSLKPEDTAVYYCAAFQWRSDDVG<br>YLNLGPLEYWGQGTQVTVSSKVSGAGGGGSMSRDQKMSQVQLQESGGGLVQAGGSLRL<br>SCAASGNISQLVYMGWYRQAPGKERELVAGITHGGITYYADSVKGRFTISRDNAKNTVYLQ<br>MNSLKPEDTAVYYCAAFQWRSDDVGYLNLGPLEYWGQGTQVTVSSKVSGAGGGGSMSR<br>DQKMSQVQLQESGGGLVQAGGSLRLSCAASGNISQLVYMGWYRQAPGKERELVAGITHG<br>GITYYADSVKGRFTISRDNAKNTVYLQMNSLKPEDTAVYYCAAFQWRSDDVGYLNLGPLEY<br>WGQGTQVTVSSKVSGA
rhx5441	BDFP1.6:1.6-6xmEosNb	This study	MSMANREVVETLKELLADGEKRVQVAGVIGTNAAEVVKTAVSLLFQEYPELVSPGGCAYTTR<br>RYNMCVRDMNYFLRMCSYAIVAGGASVLDGRMLAGFRDTFNSLGIPLCPAARGIQLMKKI<br>VKEKLATAGMTNIAFVDEPFDYIARVISETEIGHGTGSTGSGSMANREVVETLKELLADGEKR<br>VQVAGVIGTNAAEVVKTAVSLLFQEYPELVSPGGCAYTTRRYNMCVRDMNYFLRMCSYAIV<br>AGGASVLDGRMLAGFRDTFNSLGIPLCPAARGIQLMKKIVKEKLATAGMTNIAFVDEPFDYI<br>ARVISETEIGGGGSMSRDQKMSQVQLQESGGGLVQAGGSLRLSCAASGNISQLVYMGWY<br>RQAPGKERELVAGITHGGITYYADSVKGRFTISRDNAKNTVYLQMNSLKPEDTAVYYCAAFQ<br>WRSDDVGYLNLGPLEYWGQGTQVTVSSKVSGAGGGGSMSRDQKMSQVQLQESGGGLV<br>QAGGSLRLSCAASGNISQLVYMGWYRQAPGKERELVAGITHGGITYYADSVKGRFTISRDN<br>AKNTVYLQMNSLKPEDTAVYYCAAFQWRSDDVGYLNLGPLEYWGQGTQVTVSSKVSGAG<br>GGGSMSRDQKMSQVQLQESGGGLVQAGGSLRLSCAASGNISQLVYMGWYRQAPGKER<br>ELVAGITHGGITYYADSVKGRFTISRDNAKNTVYLQMNSLKPEDTAVYYCAAFQWRSDDVG<br>YLNLGPLEYWGQGTQVTVSSKVSGAGGGGSMSRDQKMSQVQLQESGGGLVQAGGSLRL<br>SCAASGNISQLVYMGWYRQAPGKERELVAGITHGGITYYADSVKGRFTISRDNAKNTVYLQ<br>MNSLKPEDTAVYYCAAFQWRSDDVGYLNLGPLEYWGQGTQVTVSSKVSGAGGGGSMSR<br>DQKMSQVQLQESGGGLVQAGGSLRLSCAASGNISQLVYMGWYRQAPGKERELVAGITHG<br>GITYYADSVKGRFTISRDNAKNTVYLQMNSLKPEDTAVYYCAAFQWRSDDVGYLNLGPLEY<br>WGQGTQVTVSSKVSGAGGGGSMSRDQKMSQVQLQESGGGLVQAGGSLRLSCAASGNI<br>SQLVYMGWYRQAPGKERELVAGITHGGITYYADSVKGRFTISRDNAKNTVYLQMNSLKPED<br>TAVYYCAAFQWRSDDVGYLNLGPLEYWGQGTQVTVSSKVSGA
rhx5092	mEos3.1	This study	MSAIKPDMKIKLRMEGNVNGHHFVIDGDGTGKPFEGKQSMDLEVKEGGPLPFAFDILTTAF<br>HYGNRVFAKYPDNIQDYFKQSFPKGYSWERSLTFEDGGICNARNDITMEGDTFYNKVRFY<br>GTNFPANGPVMQKKTLKWEPSTEKMYVRDGVLTGDVEMALLLEGNAHYRCDFRTTYKAKE<br>KGVKLPGAHFVDHCIEILSHDKDYNKVKLYEHAVAHSGLPDNARR
rhx3270	2xmEos3.1	This study	MSAIKPDMKIKLRMEGNVNGHHFVIDGDGTGKPFEGKQSMDLEVKEGGPLPFAFDILTTAF<br>HYGNRVFAKYPDNIQDYFKQSFPKGYSWERSLTFEDGGICNARNDITMEGDTFYNKVRFY<br>GTNFPANGPVMQKKTLKWEPSTEKMYVRDGVLTGDVEMALLLEGNAHYRCDFRTTYKAKE<br>KGVKLPGAHFVDHCIEILSHDKDYNKVKLYEHAVAHSGLPDNARRGGGGSSSAIKPDMKIK<br>LRMEGNVNGHHFVIDGDGTGKPFEGKQSMDLEVKEGGPLPFAFDILTTAFHYGNRVFAKY<br>PDNIQDYFKQSFPKGYSWERSLTFEDGGICNARNDITMEGDTFYNKVRFYGTNFPANGPV<br>MQKKTLKWEPSTEKMYVRDGVLTGDVEMALLLEGNAHYRCDFRTTYKAKEKGVKLPGAHF<br>VDHCIEILSHDKDYNKVKLYEHAVAHSGLPDNARR
rhx3269	3xmEos3.1	This study	MSAIKPDMKIKLRMEGNVNGHHFVIDGDGTGKPFEGKQSMDLEVKEGGPLPFAFDILTTAF<br>HYGNRVFAKYPDNIQDYFKQSFPKGYSWERSLTFEDGGICNARNDITMEGDTFYNKVRFY<br>GTNFPANGPVMQKKTLKWEPSTEKMYVRDGVLTGDVEMALLLEGNAHYRCDFRTTYKAKE<br>KGVKLPGAHFVDHCIEILSHDKDYNKVKLYEHAVAHSGLPDNARRGGGGSSSAIKPDMKIK<br>LRMEGNVNGHHFVIDGDGTGKPFEGKQSMDLEVKEGGPLPFAFDILTTAFHYGNRVFAKY<br>PDNIQDYFKQSFPKGYSWERSLTFEDGGICNARNDITMEGDTFYNKVRFYGTNFPANGPV<br>MQKKTLKWEPSTEKMYVRDGVLTGDVEMALLLEGNAHYRCDFRTTYKAKEKGVKLPGAHF<br>VDHCIEILSHDKDYNKVKLYEHAVAHSGLPDNARRGGGGSSSAIKPDMKIKLRMEGNVNG<br>HHFVIDGDGTGKPFEGKQSMDLEVKEGGPLPFAFDILTTAFHYGNRVFAKYPDNIQDYFKQ<br>SFPKGYSWERSLTFEDGGICNARNDITMEGDTFYNKVRFYGTNFPANGPVMQKKTLKWEP<br>STEKMYVRDGVLTGDVEMALLLEGNAHYRCDFRTTYKAKEKGVKLPGAHFVDHCIEILSHD<br>KDYNKVKLYEHAVAHSGLPDNARR
rhx2912	4xmEos3.1	This study	MSAIKPDMKIKLRMEGNVNGHHFVIDGDGTGKPFEGKQSMDLEVKEGGPLPFAFDILTTAF<br>HYGNRVFAKYPDNIQDYFKQSFPKGYSWERSLTFEDGGICNARNDITMEGDTFYNKVRFY<br>GTNFPANGPVMQKKTLKWEPSTEKMYVRDGVLTGDVEMALLLEGNAHYRCDFRTTYKAKE<br>KGVKLPGAHFVDHCIEILSHDKDYNKVKLYEHAVAHSGLPDNARRGGGGSSSAIKPDMKIK<br>LRMEGNVNGHHFVIDGDGTGKPFEGKQSMDLEVKEGGPLPFAFDILTTAFHYGNRVFAKY<br>PDNIQDYFKQSFPKGYSWERSLTFEDGGICNARNDITMEGDTFYNKVRFYGTNFPANGPV<br>MQKKTLKWEPSTEKMYVRDGVLTGDVEMALLLEGNAHYRCDFRTTYKAKEKGVKLPGAHF<br>VDHCIEILSHDKDYNKVKLYEHAVAHSGLPDNARRGGGGSSSAIKPDMKIKLRMEGNVNG<br>HHFVIDGDGTGKPFEGKQSMDLEVKEGGPLPFAFDILTTAFHYGNRVFAKYPDNIQDYFKQ<br>SFPKGYSWERSLTFEDGGICNARNDITMEGDTFYNKVRFYGTNFPANGPVMQKKTLKWEP<br>STEKMYVRDGVLTGDVEMALLLEGNAHYRCDFRTTYKAKEKGVKLPGAHFVDHCIEILSHD<br>KDYNKVKLYEHAVAHSGLPDNARRGGGGSSSAIKPDMKIKLRMEGNVNGHHFVIDGDGT<br>GKPFEGKQSMDLEVKEGGPLPFAFDILTTAFHYGNRVFAKYPDNIQDYFKQSFPKGYSWER<br>SLTFEDGGICNARNDITMEGDTFYNKVRFYGTNFPANGPVMQKKTLKWEPSTEKMYVRDG<br>VLTGDVEMALLLEGNAHYRCDFRTTYKAKEKGVKLPGAHFVDHCIEILSHDKDYNKVKLYEH<br>AVAHSGLPDNARR
rhx0927	ASC-mEos3.1	Khan et al., 2018	MSGRARDAILDALENLTAEELKKFKLKLLSVPLREGYGRIPRGALLSMDALDLTDKLVSFYLE<br>TYGAELTANVLRDMGLQEMAGQLQAATHQGSGAAPAGIQAPPQSAAKPGLHFIDQHRAAL<br>IARVTNVEWLLDALYGKVLTDEQYQAVRAEPTNPSKMRKLFSFTPAWNWTCKDLLLQALRE<br>SQSYLVEDLERSGAEAAAREAAAREAAAREAAARINMSAIKPDMKIKLRMEGNVNGHHFVI<br>DGDGTGKPFEGKQSMDLEVKEGGPLPFAFDILTTAFHYGNRVFAKYPDNIQDYFKQSFPKG<br>YSWERSLTFEDGGICNARNDITMEGDTFYNKVRFYGTNFPANGPVMQKKTLKWEPSTEKM<br>YVRDGVLTGDVEMALLLEGNAHYRCDFRTTYKAKEKGVKLPGAHFVDHCIEILSHDKDYNK<br>VKLYEHAVAHSGLPDNARR
rhx1111	SesA-mEos3.1	Khan et al., 2018	MSEATVSELISSTIKSLEEGIRCHKDVEDDNGLREAFHEAGRGLSVVNQALQAAQGNSARE<br>PQSAMNPLRVCNTKANISASIFKAVAQAPKTSRFEGYKEAVRQEGNGQTVEVLVVGMMKD<br>VCALAENFAIQDQVKKLHETIEKLSAMKPSLPKEGPGHTFTNIGNGNQYNATDGPQNINQG<br>GNQVTGGTFPGTVNFGSPWPQNPPFEHRNGAEAAAREAAAREAAAREAAARINMSAIKPD<br>MKIKLRMEGNVNGHHFVIDGDGTGKPFEGKQSMDLEVKEGGPLPFAFDILTTAFHYGNRV<br>FAKYPDNIQDYFKQSFPKGYSWERSLTFEDGGICNARNDITMEGDTFYNKVRFYGTNFPAN<br>GPVMQKKTLKWEPSTEKMYVRDGVLTGDVEMALLLEGNAHYRCDFRTTYKAKEKGVKLPG<br>AHFVDHCIEILSHDKDYNKVKLYEHAVAHSGLPDNARR
